# Quantitative SARS-CoV-2 Antibody Screening of Healthcare Workers in the Southern Part of Kyoto City During the COVID-19 Pre-pandemic Period

**DOI:** 10.3389/fpubh.2020.595348

**Published:** 2020-12-07

**Authors:** Kohei Fujita, Shinpei Kada, Osamu Kanai, Hiroaki Hata, Takao Odagaki, Noriko Satoh-Asahara, Tetsuya Tagami, Akihiro Yasoda

**Affiliations:** ^1^Division of Respiratory Medicine, Center for Respiratory Diseases, National Hospital Organization Kyoto Medical Center, Kyoto, Japan; ^2^Department of Infectious Diseases, National Hospital Organization Kyoto Medical Center, Kyoto, Japan; ^3^Department of Otolaryngology, Head and Neck Surgery, National Hospital Organization Kyoto Medical Center, Kyoto, Japan; ^4^Department of Surgery, National Hospital Organization Kyoto Medical Center, Kyoto, Japan; ^5^Department of General Medicine, National Hospital Organization Kyoto Medical Center, Kyoto, Japan; ^6^Department of Endocrinology and Metabolism, National Hospital Organization Kyoto Medical Center, Kyoto, Japan; ^7^Division of Endocrinology, Metabolism, and Hypertension Research, Clinical Research Institute, National Hospital Organization Kyoto Medical Center, Kyoto, Japan

**Keywords:** COVID-19, seroprevalence, SARS-CoV-2, ELISA, antibody

## Abstract

**Background:** The coronavirus disease-2019 (COVID-19) pandemic is associated with a heavy burden on the mental and physical health of patients, regional healthcare resources, and global economic activity. While understanding of the incidence and case-fatality rates has increased, there are limited data concerning seroprevalence of antibodies against the severe acute respiratory syndrome-coronavirus-2 (SARS-CoV-2) in healthcare workers during the pre-pandemic period. This study aimed to quantitatively evaluate seroprevalence of SARS-CoV-2 antibodies in healthcare workers in the southern part of Kyoto city, Japan.

**Methods:** We prospectively recruited healthcare workers from a single hospital between April 10 and April 20, 2020. We collected serum samples from these participants and quantitatively evaluated SARS-CoV-2 IgG antibody levels using enzyme-linked immunosorbent assays.

**Results:** Five (5.4%), 15 (16.3%), and 72 (78.3%) participants showed positive, borderline, and negative serum SARS-CoV-2 IgG antibody status, respectively. We found the mean titer associated with each antibody status (overall, positive, borderline, and negative) was clearly differentiated. Participants working at the otolaryngology department and/or with a history of seasonal common cold symptoms had a significantly higher SARS-CoV-2 IgG antibody titer (*p* = 0.046, *p* = 0.046, respectively).

**Conclusions:** Five (5.4%) and 15 (16.3%) participants tested positive and borderline, respectively, for SARS-CoV-2 IgG antibody during the COVID-19 pre-pandemic period. These rates were higher than expected, based on government situation reports. These findings suggest that COVID-19 had already spread within the southern part of Kyoto city at the early stage of the pandemic.

## Introduction

Coronavirus disease-2019 (COVID-19) is caused by the severe acute respiratory syndrome coronavirus-2 (SARS-CoV-2). COVID-19 was first reported in Wuhan, China, in December 2019, and the outbreak was subsequently declared a pandemic by the World Health Organization (WHO) on March 11, 2020 (1). The disease course varies from mild and self-limiting upper respiratory infection symptoms to severe respiratory failure, which might require respiratory support (2, 3). By mid-March 2020, pandemic centers were located in China, the United States, and several European countries. In Japan, the government announced a state of emergency on April 4, 2020. At the end of July 2020, >750,000 people worldwide had died of COVID-19 (1, 4). COVID-19 is associated with a heavy burden on the mental and physical health of patients, regional healthcare resources, and global economic activity. Effective policies to deal with the pandemic are required and should be founded on reliable epidemiological data. The diagnosis of COVID-19 is based on viral nucleic acid detection using a reverse-transcription polymerase chain reaction (RT-PCR) assay for SARS-CoV-2. Whereas, an RT-PCR assay is accurate at detecting an active case of COVID-19, identifying individuals who have recovered from SARS-CoV-2 infection has been challenging. In contrast to tracking active cases, antibody detection can provide information on individual and herd-acquired immunity against SARS-CoV-2. Furthermore, an antibody assay can help to estimate the number of people within a community who remain potential cases, assisting governments in effective decision-making. To date, data concerning the seroprevalence of SARS-CoV-2 antibodies in healthcare workers worldwide are limited. During the pre-pandemic period, we quantitatively evaluated the seroprevalence of SARS-CoV-2 antibodies in healthcare workers in the southern part of Kyoto city, an area famous for its heritage status and a popular tourist destination.

## Participants and Methods

### Participants

This study was conducted at the National Hospital Organization Kyoto Medical Center (600 beds), located in southern Kyoto, Japan. In response to the pandemic, our hospital formed an infectious disease department dedicated to COVID-19, involving medical staff such as internal medicine physicians, chest physicians, general and thoracic surgeons, cardiologists, nephrologists, otolaryngologists, and emergency physicians. We prospectively recruited medical doctors, nurses, and ward clerks employed at our hospital between April 10 and April 20, 2020. All participants were asymptomatic and worked within any of the following departments: infectious disease, respiratory medicine, otolaryngology, or emergency medicine. Healthcare workers from these departments were selected as they were considered more likely to treat patients with suspected COVID-19, of which they might not have been aware. Additionally, we collected the following questionnaire-based data: a history of seasonal common cold from winter 2019 to early spring 2020 and a history of regular contact with children aged <12 years. The questionnaires were created based on previous studies involving behavior patterns during the H10N8 avian influenza outbreak (5).

### ELISA Assay

We collected 6 ml of blood from each participant between April 10 and April 20, 2020. After extracting serum, we deep froze and stored the samples at −80°C. We used an enzyme-linked immunosorbent (ELISA) assay, using COVID-19 IgG ELISA kits (DRG international, Inc. Springfield, NJ, USA), to evaluate the presence of serum IgG antibody against SARS-CoV-2, in accordance with the manufacturer's instructions. Briefly, 1:100 diluted human serum samples were placed onto a 96-well microplate (coated with SARS-CoV-2 recombinant full-length nucleocapsid protein) and then incubated for 30 min at room temperature (20–25°C). After washing, 100 μl HRP-labeled anti-IgG tracer antibody was added into the wells and the samples were incubated for 30 min at room temperature (20–25°C). Following the second wash cycle, 100 μl substrate was added into the wells and the samples were incubated for 20 min at room temperature (20–25°C). Last, stop solution was added into the wells to terminate the reaction. The optical density of each well was determined using a microplate reader set to 450 nm within 10 min. For IgG detection, the cut-off value was modified through using an internal negative and positive control of Japanese samples, because of the differences in ethnicity between ELISA kits (Chinese controls) and our samples (Japanese). We interpreted the results as positive, borderline, and negative, according to the manufacturer's instructions.

### Statistical Analysis

The data were analyzed using JMP version 14.0.0 (SAS institute Inc. Cary, NC). A Fisher's exact test was used to compare proportions among occupations, wards, questionnaires, and SARS-CoV-2 IgG antibody status. Wilcoxon rank sum or Kruskal-Wallis tests, as appropriate, were used to compare SARS-CoV-2 IgG antibody titers between groups, and *p*-values < 0.05 were considered statistically significant.

### Ethical Approval

This study was approved by the relevant institutional review boards (approval number: 20-009) and written informed consent was obtained from all study participants.

## Results

In total, 92 healthcare workers were recruited for this study. Medical doctors, nurses, and medical clerks comprised 42 (45.7%), 48 (52.2%), and 2 (2.2%) participants, respectively. Of 92 participants, 59 (64.1%) were women, and most participants were aged between 20 and 39 years. Among the participants, the otolaryngology department was the most common place of work, followed by the respiratory and emergency medicine departments. Of 92 participants, 47 (51.1%) had a history of seasonal common cold symptoms from winter 2019 to early spring 2020, and 19 (20.7%) participants had a history of regular contact with children aged <12 years (Table 1).

**Table 1:** Clinical and demographic characteristics of participating healthcare workers.

	***n* = 92**
Sex (female)	59 (64.1)
**Age group (years)**	
20–29	30 (32.6)
30–39	29 (31.5)
40–49	21 (22.8)
≥50	12 (13.0)
**Occupation**	
Medical doctor	42 (45.7)
Nurses	48 (52.2)
Medical clerk	2 (2.2)
**Department**	
Department of Infectious Diseases	18 (19.6)
Respiratory Medicine Ward	22 (23.9)
Otolaryngology Ward	30 (32.6)
Emergency Medicine Ward	22 (23.9)
**Questionnaire**	
^*^History of seasonal common cold symptoms	47 (51.1)
^*^History of regular contact with children	19 (20.7)
^**^History of exposure to a viral infection	84 (91.3)
**SARS-CoV-2 antibody status**	
Positive	5 (5.4)
Borderline	15 (16.3)
Negative	72 (78.3)

*Data are shown as counts (%). SARS-CoV-2, severe acute respiratory syndrome coronavirus-2*.

* *Covered period from winter 2019 to early spring 2020, including history of regular contact with children aged <12 years*.

** *Participants considered exposed to viral infection were defined as those with their own history of seasonal common cold symptoms and/or examining outpatients with common cold symptoms*.

### Seroprevalence of Antibodies Against SARS-CoV-2

In total, 92 serum samples collected between April 10 and April 20, 2020 were tested for antibodies against SARS-CoV-2. Of 92 participants, 5 (5.4%), 15 (16.3%), and 72 (78.3%) showed positive, borderline, and negative SARS-CoV-2 IgG antibody test results, respectively (Table 1). There were no significant differences in antibody status between the professional groups (Table 2). We identified 2 and 3 participants with a positive antibody status in the respiratory disease and otolaryngology departments, respectively. The highest proportion of participants with a positive and borderline SARS-CoV-2 IgG antibody status worked at the otolaryngology department, whereas the lowest proportion were working at the emergency medicine department (Table 3).

**Table 2:** SARS-CoV-2 IgG antibody seroprevalence among healthcare workers according to occupation.

		**IgG against SARS-CoV-2**						***p*-value**
**Occupation**	***n***	**Positive**		**Borderline**		**Negative**		
Medical doctor	42	2	(4.7%)	6	(14.0%)	34	(76.2%)	0.9236
Nurse and Medical clerk	50	3	(6.0%)	9	(18.0%)	38	(76.0%)	

*Data are shown as counts (%). The p-value was estimated using the Fisher's exact test, with p < 0.05 considered statistically significant*.

*SARS-CoV-2, severe acute respiratory syndrome coronavirus-2*.

**Table 3:** SARS-CoV-2 IgG antibody seroprevalence among healthcare workers according to department.

		**IgG against SARS-CoV-2**						***p*-value**
	***n***	**Positive**		**Borderline**		**Negative**		
Department of Infectious	18	0	(0.0%)	3	(16.7%)	15	(83.3%)	0.2102
Diseases								
Respiratory Diseases Ward	22	2	(9.1%)	4	(18.2%)	16	(72.7%)	
Otolaryngology Ward	30	3	(10.0%)	7	(23.3%)	20	(66.7%)	
Emergency Medicine Ward	22	0	(0.0%)	1	(4.6%)	21	(95.5%)	

*Data are shown as counts (%). The p-value was estimated using a Fisher's exact test, with p < 0.05 considered statistically significant*.

*SARS-CoV-2, severe acute respiratory syndrome coronavirus-2*.

Participants with a history of seasonal common cold from winter 2019 to early spring 2020 showed a higher rate of positive SARS-CoV-2 IgG antibody test results than participants with no such history (*p* = 0.046). A history of regular contact with children or of exposure to a viral infection did not affect the seroprevalence of SARS-CoV-2 IgG antibody (Table 4).

**Table 4:** Seroprevalence of SARS-CoV-2 IgG antibody according to exposure status as determined using a questionnaire.

		**IgG against SARS-CoV-2**						***p*-value**
	***n***	**Positive**		**Borderline**		**Negative**		
^*^History of seasonal common cold symptoms	47	5	(10.6%)	9	(19.2%)	33	(70.2%)	0.0458
^*^History of regular contact with children	19	1	(5.3%)	1	(5.3%)	17	(89.5%)	0.3294
^**^History of exposure to a viral infection	84	5	(6.0%)	14	(16.7%)	65	(77.4%)	>0.99

*Data are shown as counts (%). The p-value was estimated using a Fisher's exact test, with p < 0.05 considered statistically significant*.

*SARS-CoV-2, severe acute respiratory syndrome coronavirus-2*.

* *Covered period from winter 2019 to early spring 2020, including history of regular contact with children aged <12 years*.

** *Participants considered exposed to viral infection were defined as those with own history of seasonal common cold symptoms and/or examining outpatients with common cold symptoms*.

### Serum SARS-CoV-2 IgG Antibody Titer

**Figure 1:**
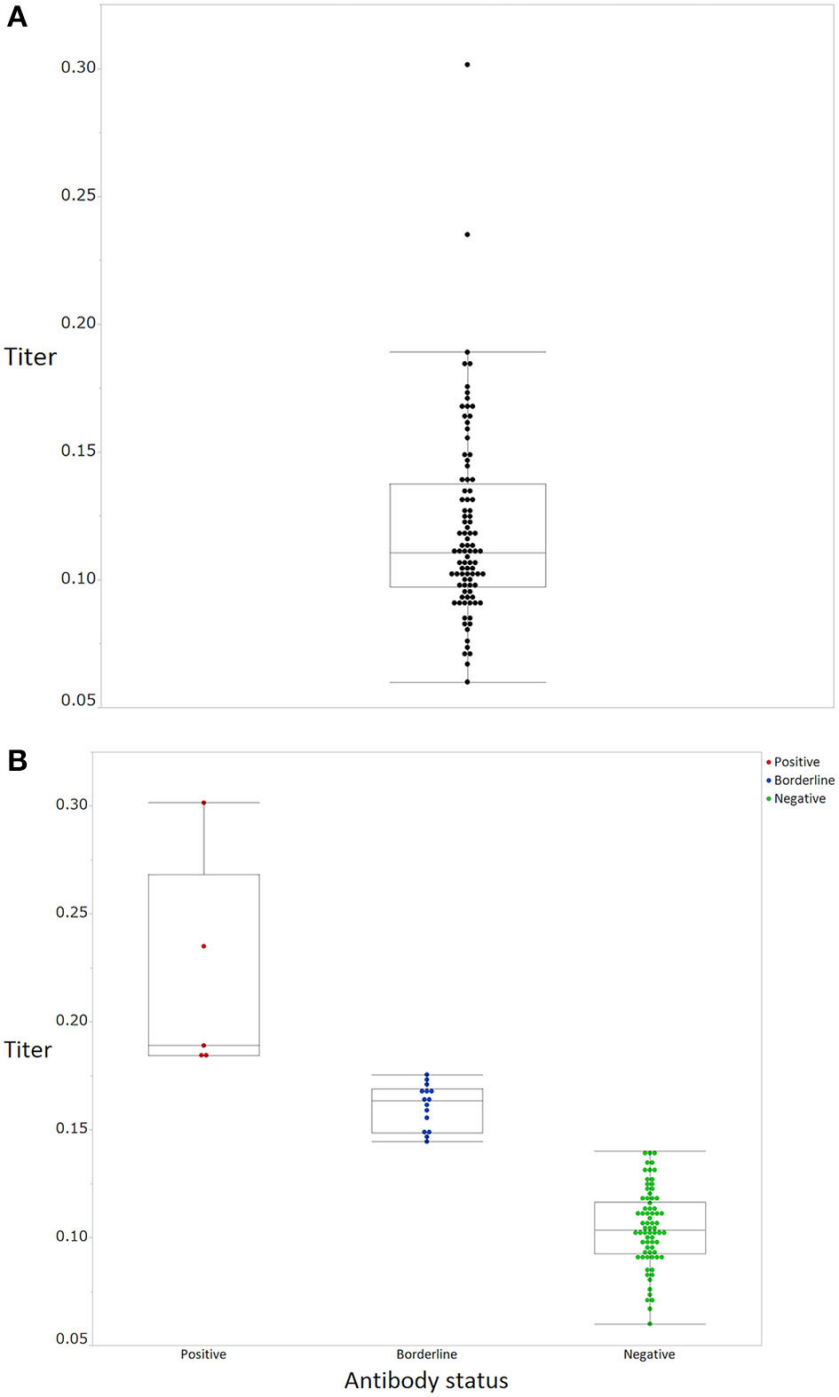
SARS-CoV-2 IgG antibody titers of the participants. **(A)** All participants (black), **(B)** stratified according to positive (red), borderline (blue), and negative (green) status. Boxes correspond to the interquartile range of values for each group; error bars show the 90th percentile range.

**Figure 2:**
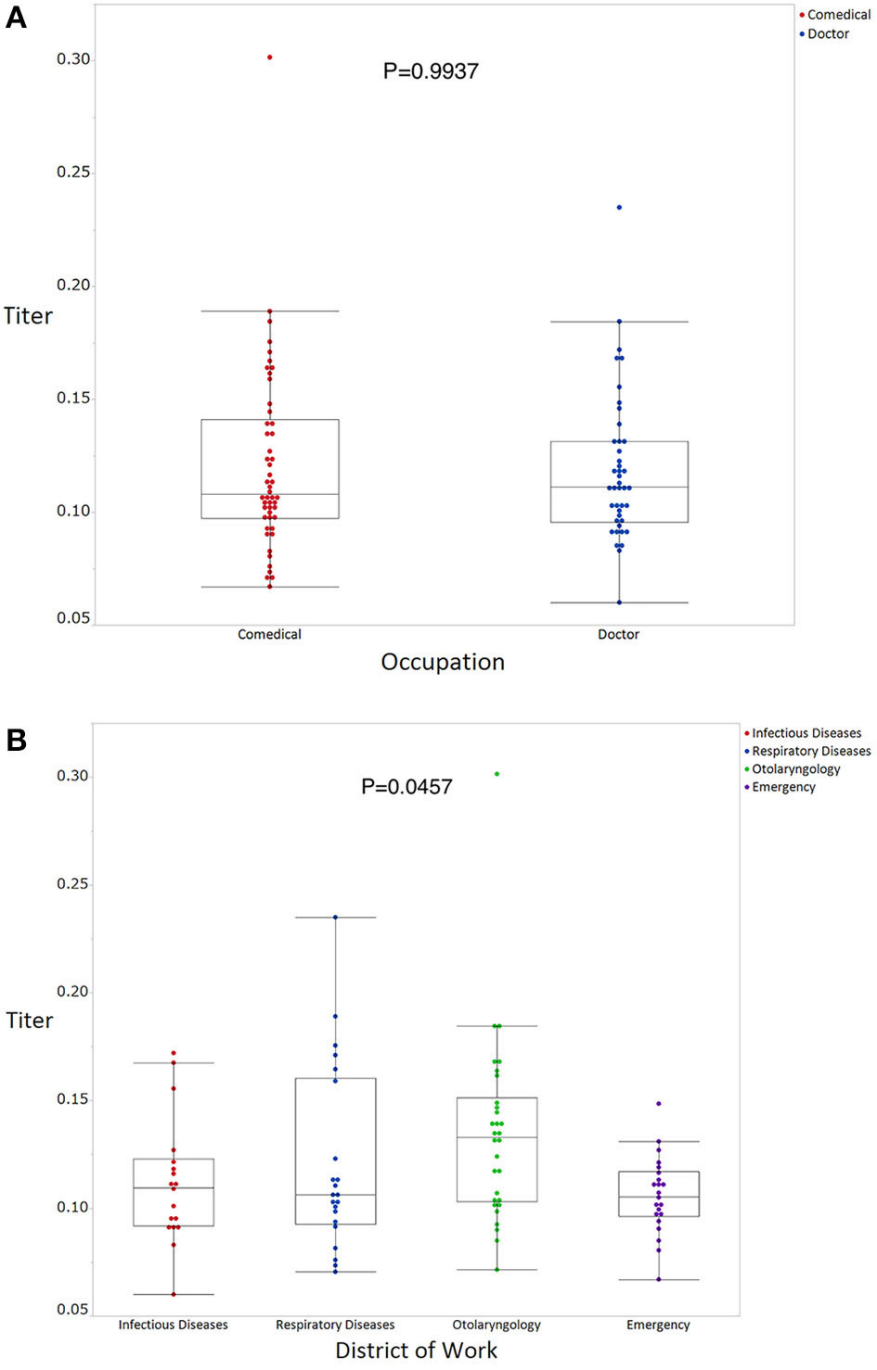
SARS-CoV-2 IgG antibody titers stratified according to occupation **(A)** and department **(B)**. A Wilcoxon rank sum test **(A)** or a Kruskal-Wallis test **(B)** was used to compare the titer of SARS-CoV-2 IgG antibody levels between the groups, with *p*-values < 0.05 considered statistically significant.

**Figure 3:**
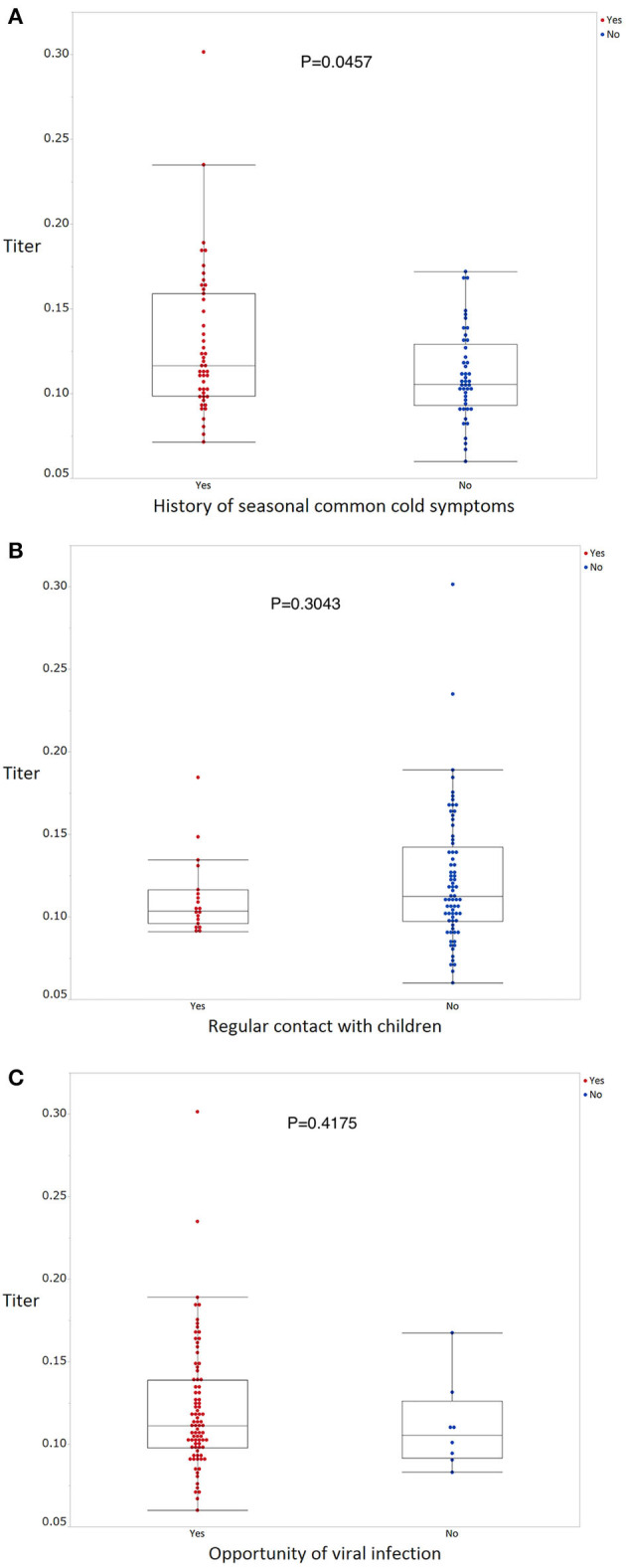
SARS-CoV-2 IgG antibody titers stratified according to exposure status as determined using questionnaires concerning behavior patterns **(A–C)**. Wilcoxon rank sum tests were used to compare the titer of SARS-CoV-2 IgG antibody levels between the groups, with *p*-values < 0.05 considered statistically significant.

The mean antibody titer of all participants was 0.120 ± 0.0372 (Figure 1A). The mean titer of the antibody positive, borderline, and negative groups was 0.219 ± 0.051, 0.161 ± 0.0101, and 0.105 ± 0.018, respectively (Figure 1B). Mean antibody titers stratified according to occupation and department are shown in Figures 2A,B. There were no significant differences in mean antibody titers between doctors, nurses, and medical clerks (0.119 ± 0.0326 and 0.121 ± 0.0058, *p* = 0.994; Figure 2A). The mean antibody titer among workers at the otolaryngology department was significantly higher than that among workers in the other three departments (0.112 ± 0.029, 0.121 ± 0.043, 0.134 ± 0.043, and 0.11 ± 0.018, *p* = 0.046; Figure 2B). Participants with a history of seasonal common cold symptoms had a significantly higher titer of SARS-CoV-2 IgG antibody than those with no such history (0.13 ± 0.044 and 0.11 ± 0.026, *p* = 0.046; Figure 3A). There were no significant differences in the mean antibody titer between participants with and without a history of regular contact with children or with a history of exposure to a viral infection (*p* = 0.304, Figure 3B; *p* = 0.418, Figure 3C).

## Discussion

In this study, 5 (5.4%) and 15 (16.3%) healthcare workers were positive and borderline, respectively, for the presence of SARS-CoV-2 IgG antibodies. The mean antibody titer among the borderline group was clearly distinct from that of the negative group. Participants with borderline antibody results might have been latently sensitized by patients with COVID-19. As our hospital accepted patients with confirmed COVID-19 after April 15, 2020, the antibody status of our study participants might reflect community-acquired immunity, resulting from unwitting exposure in daily medical practice.

The mean antibody titer was significantly higher among workers at the otolaryngology department than among those working in other departments. This suggests that healthcare workers within the otolaryngology department were more likely to be exposed to SARS-CoV-2 than their counterparts working in other departments.

According to official statements from Kyoto city authorities, confirmed incidence and fatality associated with COVID-19 in Kyoto city at the end of April 2020 comprised 215 and 11 cases, respectively (6). However, antibody seroprevalence observed in this study was much higher than that expected, based on data from government reports. Furthermore, the number of participants with borderline antibody status in our study was nearly 3 times that of participants with a positive antibody status.

Recently, several studies have reported that the population-wide seroprevalence of SARS-CoV-2 antibodies was higher than expected, based on the number of confirmed cases (7, 8). For example, in Kobe city, Japan, 3.3% of outpatients tested SARS-CoV-2 IgG antibody positive (8). Using quantitative methods, our results showed there were >3 times the number of people sensitized with SARS-CoV-2 than those found to have positive SARS-CoV-2 IgG antibody. Given that the pathogenicity of SARS-CoV-2 appears to be similar to SARS-CoV, most patients infected with SARS-CoV-2 will express specific IgG antibodies within 1 week−3 months after infection (9). In light of this timeline of seroconversion, the study participants with a positive or borderline antibody status were likely to have been exposed to SARS-CoV-2 between December 2019 and March 2020. These findings suggest that COVID-19 was already present in Kyoto at the early stages of pandemic. The period between December and March is a time of heightened tourist activity in Kyoto, in particular, involving tourists from China and Taiwan who are celebrating the Chinese New Year spring festival. After the spring festival, on March 5, 2020, the Japanese government implemented a strict ban on travelers arriving from China.

According to epidemiological data provided by the WHO (1) and by Johns Hopkins University (4), incidence and case-fatality rates in major European countries (Germany, United Kingdom, France, Italy, and Spain) and the United States are much higher than those in major Asian countries (China, Japan, South Korea, and Taiwan). There are several possible explanations for this phenomenon. Differences in lifestyle and behavioral habits between Western and Asian populations might explain some of the variability in these rates. Some studies have shown a correlation between universal BCG vaccination policy, and morbidity and mortality associated with COVID-19 (10, 11). Although this hypothesis has resulted in clinical trials to evaluate the efficacy of BCG vaccination against COVID-19 (NCT04327206 and NCT04362124), restricted basic and clinical evidence makes this association difficult to evaluate. Meanwhile, other authors have suggested that differences in viral genotypes and virulence may affect morbidity and mortality associated with COVID-19; however, this explanation requires further elaboration. The National Institute of Infectious Diseases of Japan reported that the first wave of the COVID-19 pandemic emerged from Wuhan, China, and flattened toward the end of March; however, a second wave emerged from European countries, spreading across Japan after the end of March (12). Nevertheless, even during the second wave, Japan retained its much lower morbidity and mortality rates compared to those of Western countries, as reported at the end of April 2020 (13). Kamikubo and Takahashi hypothesized that the pre-pandemic spread of a low-virulence type of SARS-CoV-2 and subsequent exposure to a mild-virulence type of SARS-CoV-2 induced herd immunity, which reduced the severity of a high-virulence type of SARS-CoV-2 in Japan (14). The findings concerning borderline antibody titers in the present study offer some support for this hypothesis. In this study, participants with a history of seasonal common cold from winter 2019 to early spring 2020 had a significantly higher SARS-CoV-2 antibody titer than those with no such history, which might have resulted from pre-pandemic exposure to low- and middle-virulence types of SARS-CoV-2. Importantly, a history of exposure to seasonal cold should not be interpreted as equivalent to a history of subclinical exposure to SARS-CoV-2 virus, since this virus was supposedly absent from the environment during the last season of the common cold. Further research is required to verify this hypothesis.

This study had several limitations. First, this was a single-center study; therefore, selection bias might have affected our findings. Second, the small sample size restricted the statistical power of our analyses. Third, as our participants were recruited from departments where exposure to COVID-19 was more likely, the reported seroprevalence might be an overestimate relative to that of workers in other departments or within the general population. Fourth, because the COVID-19 pandemic is ongoing, a significant proportion of available research results that we have referred to might be premature. Fifth, we did not evaluate the IgM antibody. A negative serum for IgG antibody might nevertheless contain a specific IgM antibody, especially as samples were obtained at the early stages of the pandemic. The interpretation of these results would likely be affected by the quantitation of IgM.

In conclusion, our study findings indicated a relatively high frequency of healthcare workers with a positive or borderline SARS-CoV-2 antibody status in the southern part of Kyoto city, an area frequented by tourists. Our results suggest that COVID-19 might already have been present in Kyoto at the early stage of the pandemic. Several previous studies have evaluated SARS-CoV-2 antibody profiles in patients with COVID-19 (15–17); however, our study is the first to quantitatively evaluate antibody levels in healthcare workers involved with patients during the COVID-19 pre-pandemic period. Serial evaluation of SARS-CoV-2 IgG antibody status is likely to reveal risk factors associated with COVID-19 susceptibility and mechanisms of disease spread. Finally, these results should be approached with caution, as there remains a lack of evidence regarding the role of antibodies present after recovery from COVID-19 in developing immunity against subsequent infections.

## Data Availability Statement

The raw data supporting the conclusions of this article will be made available by the authors, without undue reservation.

## Ethics Statement

The studies involving human participants were reviewed and approved by NHO Kyoto Medical Center IRB. The patients/participants provided their written informed consent to participate in this study.

## Author Contributions

KF and SK designed the study and collected blood samples. KF, SK, OK, HH, and TO recruited the participants and collected the data. TT, NS-A, and AY managed the ELISA tests and interpreted the results. KF, SK, and OK participated in the statistical analysis. KF and SK drafted and revised the manuscript. All authors contributed to the article and approved the submitted version.

## Conflict of Interest

The authors declare that the research was conducted in the absence of any commercial or financial relationships that could be construed as a potential conflict of interest.
